# A longitudinal study of women’s memories of their childbirth experiences at five years postpartum

**DOI:** 10.1186/1471-2393-14-221

**Published:** 2014-07-05

**Authors:** Kenji Takehara, Makiko Noguchi, Takuya Shimane, Chizuru Misago

**Affiliations:** 1 Department of Health Policy, National Center for Child Health and Development, 10-1, Okura 2chome, Setagaya, Tokyo 157-8535, Japan; 2 Faculty of Health Sciences, Hokkaido University, N12-W5, Kita-ku, Sapporo 060-0812, Japan; 3 Department of Drug Dependence Research, National Center of Neurology and Psychiatry, 4-1-1 Ogawahigashi, Kodaira, Tokyo 187-8553, Japan; 4 Department of International and Cultural Studies, Tsuda College, 2-1-1 Tsuda-machi, Kodaira-shi, Tokyo 187-8577, Japan

## Abstract

**Background:**

Few studies have investigated whether women can accurately recall their birthing experiences after a long period. We investigated the consistency of women’s memories of their childbirth experiences between those at a few days postpartum and 5 years later.

**Methods:**

This prospective cohort study comprised 1,168 women who delivered at a maternity hospital and four maternity homes in Japan between May 2002 and August 2003. Data were collected using structured interviews and transcriptions from medical records. The childbirth experience was assessed using the Childbirth Experience Scale (CBE-Scale) at a few days postpartum and 5 years later.

**Results:**

We obtained 584 (50.0%) valid responses from women who completed the survey at a few days postpartum and 5 years later. Significant differences were observed in 16 out of 18 items on the CBE-Scale when responses were compared at both time points. Women who answered "yes" to any item on the CBE-Scale at the baseline survey tended to demonstrate a more precise recollection for that item 5 years after childbirth than those who answered "no" for the corresponding item.

**Conclusions:**

We conclude that women remember their childbirth experience clearly at 5 years after the childbirth.

## Background

Childbirth experience has a powerful effect on women and the well-being of their babies with the potential for permanent or long-term impact, both positive and negative [1-3]. Several previous studies have investigated the effects of the childbirth experience on women’s lives. Although these studies should be conducted on the assumption that women clearly remember their childbirth experience, we do not know whether women remember their childbirth experience clearly, and if they do, for how long.

The few studies evaluating a mother’s memory of experiencing labor have focused on two major aspects: labor pain and childbirth. The body of literature regarding recollection of labor pain is well represented in a review article, wherein labor pain and duration of recall were assessed via a literature search of articles published between 1990 and 1999 [4], and a well-designed prospective cohort study, in which 1,383 women were followed up to 5 years after childbirth [5]. Taken together, the conclusion emerging from these studies is that women tend to remember labor pain, but forget the details over time.

Studies regarding memories of the childbirth experience are even more limited [6,7]; these studies focused either on how positive or negative experiences influence women’s lives or on the determinants of their childbirth experience, including the mother–midwife and mother–partner relationships, and maternal depression. Waldenstrom concluded that, on average, the overall memory of the birth experience became more negative over time [7]. On the other hand, Stadlmayr et al. showed that some aspects of women’s memories of childbirth experience generally improve with time [6]. Rijnders et al. conducted a study of women’s recall of childbirth at 3 years postpartum, and showed that most women looked back positively on their birth experience [8]. Owing to the contradictory results and small sample sizes observed in these studies, it is difficult to establish a strong conclusion concerning the consistency of women’s memories of the childbirth experience. In this study, we investigated the consistency of women’s memories of their childbirth experiences at a few days postpartum and 5 years later.

## Methods

### Subjects

We analyzed a set of data from a prospective closed cohort study conducted in Japan that investigated the impact of women’s childbirth experience on their lives, mother-child relationships, and the development of their children. All women who delivered at a maternity hospital and 4 maternity homes in Japan between May 2002 and August 2003 were asked to participate in the study by one of the interviewers who were specifically trained for this study at the facility. Women who could not communicate in Japanese or who had severe health complications after the childbirth were excluded from the study at the discretion of the head of the facility. A total of 2,314 women were recruited at a few days after childbirth and 1,453 agreed to answer the baseline questionnaire survey; subsequently, 1,168 women agreed to participate in the follow up study. The interviewers arranged an appointment with each participant for a structured interview as part of the baseline survey. Most participants agreed to conduct the interview immediately after recruitment.

### Instrument

Socio-demographic data were collected using a structured questionnaire. The childbirth experience scale (CBE-Scale), which consists of four factors ("happiness", "body sense", "discovery", and "unaffected self expression") and 18 items was used to assess information regarding childbirth experience both at baseline and at 5 years postpartum. Participants answered these questions with either "yes" or "no", depending on whether they experienced the specific questionnaire item during childbirth, or not. The CBE-Scale was tested for reliability and validity in the same cohort study [9]. The acceptance of an interview or the return of questionnaires completed by the participants was regarded as consent to participate in this study. Age, place of birth, and data regarding the labor and birthing experience were also collected from medical records.

### Ethical considerations

After gaining verbal consent from the women and the heads of the facilities, the interviewers transcribed personal and medical information from medical records. All study participants also provided verbal consent to participate in this cohort study before each follow-up survey was conducted. This study was approved by the Ethical Committee of the National Center for Child Health and Development in Japan.

### Data collection

The participants were followed and administered questionnaires seven times: at 4 months, 9 months, 16 months, 2.5 years, 3 years, 3.5 years, and 5 years after childbirth. In the baseline survey, basic characteristics, childbirth experience, medical intervention and the health condition in the postpartum period were measured. At the first and second follow-up surveys, child development and maternal depression were measured, Child temperament and child-rearing anxiety were assessed at the third follow up, while parental bonding, childcare stress, and child behavior were assessed at the forth and fifth follow-ups. Allergies and results of a health checkup at 3 years old were assessed at the sixth follow-up. Finally, at the seventh follow-up, childbirth experience was assessed again along with the mother’s working environment. We used the baseline data and seventh follow-up survey data for this study.

The seventh follow-up survey, at 5 years after childbirth, was carried out with women who participated in the sixth follow-up survey, which was conducted at 3.5 years after childbirth. Efforts were made to assign the same interviewer who conducted the baseline survey to the seventh follow-up survey. The interviewer arranged an appointment with the woman to conduct a structured interview using a questionnaire. When an interviewer was not able to make contact with the woman, the survey was conducted by telephone or by mail.

The seventh follow-up questionnaire included all items of the CBE-Scale to make a comparison with the answers from the CBE-Scale between the baseline survey and the seventh follow-up surveys. Women were asked to choose an answer regarding their memory of their childbirth experience from three options: "yes", "no", and "I do not remember".

### Data analysis

A two-sample test for equality of proportion with continuity correction was used to examine the associations between responses to each item of the CBE-Scale at the baseline and follow-up surveys. The test was conducted to compare two proportions for which the responses between two points of the survey were different. The responses to each item on follow-up surveys were converted into dichotomous variables depending on whether respondents remembered their childbirth experience accurately. Thus, participants who answered, "I don’t remember" on a follow-up survey were included in both the numerator and the denominator when the proportion of each group was calculated.

Spearman’s rank-order correlation coefficients between CBE-Scale score (range 0–18) and each factors were calculated both at baseline and 5 years postpartum. Partial correlation analysis was conducted to examine the factors at baseline that might influence women’s memory of childbirth, such as maternal depression at 4 months after the childbirth as well as subsequent childbirth and return to work, for a period of 5 years after the childbirth.

Statistical analysis was performed using IBM SPSS Statistics 19. All reported p values are two-tailed and not adjusted for multiple testing.

## Results

A total of 1,168 women agreed to participate in this cohort study and completed the baseline survey, and 540 (46.2%) women dropped out of the study by the sixth follow-up survey at 3.5 years postpartum. For the seventh follow-up survey at 5 years postpartum, we contacted 628 (53.8%) participants who had participated in the sixth follow-up survey and obtained 584 (50.0%) valid responses. Of these, 524 (89.7%) women reported that the follow-up survey was conducted by interviewers who had not previously interviewed them. For the follow-up survey, interviewers collected data from 324 (55.5%) women by interview and from 260 (44.5%) by mail. Homogeneity of variance was tested using Levene’s test due to concern regarding potential selection bias, as 50% of participants had dropped out by the seventh postpartum survey. The homogeneity of variance was assessed to determine whether there were differences in baseline CBE-Scale score variability between women who dropped out prior to the seventh follow-up and those who remained in the study; the results indicated that there was no difference between these groups (F = 0.072, P = 0.79).

Of the 584 participants in this study, 230 (39.4%) delivered in one of the maternity homes. At the baseline survey, the mean age of all participants was 31.9 years, ranging from 17 to 42 years. Of the participants, 162 (27.2%) were college or university graduates, or had additional education. Furthermore, 258 (44.2%) women were primiparous and 326 (55.8%) women were multiparous; 205 (35.5%) women experienced the subsequent childbirth within the 5-year study period (Table 1).

**Table 1:** Subject characteristics according to reproductive history

		**Primiparous (N = 258)**		**Multiparous (N = 326)**	
		**n**	**(%)**	**n**	**(%)**
**Sociodemographic data at the time of childbirth**					
Age (yr)					
	< 25	19	(7.4)	4	(1.2)
	25–35	200	(77.5)	246	(75.5)
	> 35	39	(15.1)	76	(23.3)
Marital status					
	Married	255	(98.8)	324	(99.4)
	Single	3	(1.2)	2	(0.6)
Place of birth					
	Maternity home	72	(27.9)	158	(48.5)
	Hospital	186	(72.1)	168	(51.5)
Education					
	Junior high or high school	65	(25.2)	96	(29.4)
	Junior college or vocational school	123	(47.7)	138	(43.3)
	College or university and above	70	(27.2)	92	(28.2)
House income per year (JPY)					
	≤ 3 million	13	(5.5)	24	(7.9)
	3–5 million	63	(26.4)	80	(26.3)
	5–10 million	144	(60.5)	164	(53.8)
	≥ 10 million	18	(7.6)	37	(12.1)
Return to work after childbirth					
	Yes	116	(45.5)	182	(56.2)
	No	139	(54.5)	142	(43.8)
**Labor and birth data**					
Mode of delivery					
	Normal vaginal	196	(76.0)	289	(88.7)
	Instrumental vaginal	24	(9.3)	8	(2.4)
	Elective cesarean	18	(7.0)	22	(6.7)
	Emergency cesarean	20	(7.8)	7	(2.1)
Referral					
	None	255	(98.8)	325	(99.7)
	During labor	2	(0.8)	0	(0.0)
	After childbirth	1	(0.4)	1	(0.3)
Birth control					
	Yes	150	(58.1)	165	(50.6)
	No	108	(41.9)	161	(49.4)
Subsequent childbirth					
	Yes	133	(52.2)	72	(22.3)
	No	122	(47.8)	251	(77.7)

Tables 2, 3, 4 and 5 show the results of the two-sample test for equality of proportion comparing women’s responses on the CBE-Scale obtained a few days postpartum to those from 5 years later. There were significant differences in 16 out of 18 items on the CBE-Scale when responses to the same items on both surveys were compared. The percentages of women whose responses at the baseline survey corresponded with those at 5 years postpartum ranged from 57.5% to 87.4% for all items of the CBE-Scale. In particular, the concordance rates for all items of the "unaffected self expression factor" were over 80%. There was a greater coincidence between surveys at baseline and 5 years after childbirth for women who answered "yes" to an item on the CBE-Scale than for those who answered "no" to the same item. The difference in the proportion of women who did not remember their childbirth experience accurately in the two groups was over 50% for eight items (Item number 7, 8, 11, 12, 14, 16–18). The percentage of responses for a particular item in the follow-up survey that did not match those of the baseline was as high as 42.5% (Item number 13). Table 6 shows that the total score and each factor score for the CBE-Scale at baseline significantly correlated with the score at 5 years after childbirth (ρ = 0.44; P > 0.01). Similarly, the total and each factor score also significantly correlated after stratifying by subsequent childbirth and return to work after childbirth. There was no significant distinction between women who experienced subsequent childbirth and a return to work and those who did not for most correlation coefficient.

**Table 2:** Responses on the "happiness" factor of the CBE-Scale at baseline and 5 years postpartum

**Response after childbirth**	**Response after 5 years**						**Two-sample test**	**Concordance rate**
	**Yes**		**No**		**I don’t remember**			
	**n**	**(%)**	**n**	**(%)**	**n**	**(%)**		
1. Was childbirth enjoyable?								
Yes	207	(85.5)	35	(14.5)	0	(0.0)	< 0.001	58.3%
No	204	(59.8)	133	(39.0)	4	(1.2)		
2. Did the childbirth feel good?								
Yes	147	(78.6)	37	(19.8)	3	(1.6)	< 0.001	65.2%
No	157	(39.9)	232	(58.9)	5	(1.3)		
3. Did you feel happy during the childbirth?								
Yes	216	(79.7)	52	(19.2)	3	(1.1)	< 0.001	59.1%
No	179	(57.6)	128	(41.2)	4	(1.3)		
4. Immediately after childbirth, did you feel that you wanted to have another child?								
Yes	99	(68.8)	41	(28.5)	4	(2.8)	= 0.658	66.9%
No	144	(32.8)	291	(66.3)	4	(0.9)		

**Table 3:** Responses on the "body sense" factor of the CBE-Scale at baseline and 5 years postpartum

**Response after childbirth**	**Response after 5 years**						**Two-sample test**	**Concordance rate**
	**Yes**		**No**		**I don’t remember**			
	**n**	**(%)**	**n**	**(%)**	**n**	**(%)**		
5. Did you feel that you were in control during childbirth?								
Yes	187	(77.3)	53	(21.9)	2	(0.8)	< 0.001	58.3%
No	155	(55.6)	117	(41.9)	7	(2.5)		
6. Did you feel that you had your own pace and rhythm during childbirth?								
Yes	260	(74.9)	84	(24.2)	3	(0.9)	< 0.001	65.6%
No	89	(50.6)	83	(47.2)	4	(2.3)		
7. Were you able to believe in yourself during childbirth?								
Yes	375	(93.3)	21	(5.2)	6	(1.5)	< 0.001	77.9%
No	85	(69.7)	33	(27.0)	4	(3.3)		
8. Were you able to express yourself in an honest way during the childbirth?								
Yes	457	(92.1)	39	(7.9)	0	(0.0)	< 0.001	84.3%
No	48	(56.5)	33	(38.8)	4	(4.7)		
9. Did you understand what was happening to your body during the childbirth?								
Yes	355	(84.5)	64	(15.2)	1	(0.2)	< 0.001	75.0%
No	63	(60.6)	38	(36.5)	3	(2.9)		
10. Were you at ease during the childbirth?								
Yes	167	(72.6)	60	(26.1)	3	(1.3)	= 0.021	67.0%
No	109	(36.9)	185	(62.7)	1	(0.3)		

**Table 4:** Responses on the "discovery" factor of the CBE-Scale at baseline and 5 years postpartum

**Response after childbirth**	**Response after 5 years**						**Two-sample test**	**Concordance rate**
	**Yes**		**No**		**I don’t remember**			
	**n**	**(%)**	**n**	**(%)**	**n**	**(%)**		
11. Did you feel that through childbirth you encountered a part of yourself that you did not previously know?								
Yes	353	(86.1)	54	(13.2)	3	(0.7)	< 0.001	71.3%
No	105	(61.0)	62	(36.0)	5	(2.9)		
12. Did you feel that childbirth meant looking within yourself and searching for insight?								
Yes	364	(87.7)	45	(10.8)	6	(1.4)	< 0.001	72.6%
No	108	(63.9)	60	(35.5)	1	(0.6)		
13. During childbirth, did you feel that you had no boundaries?								
Yes	121	(58.5)	73	(35.3)	13	(6.3)	= 0.797	57.5%
No	116	(37.3)	177	(56.9)	18	(5.8)		
14. Did you feel that a higher power was at work and that you were being moved by it?								
Yes	268	(84.3)	45	(14.2)	5	(1.6)	< 0.001	63.2%
No	135	(66.2)	62	(30.4)	7	(3.4)		
15. Were any of your actions during childbirth completely unexpected even to yourself?								
Yes	140	(68.0)	62	(30.1)	4	(1.9)	= 0.018	61.5%
No	125	(39.6)	181	(57.3)	10	(3.2)		

**Table 5:** Responses on the "unaffected self-expression" factor of the CBE-Scale at baseline and 5 years postpartum

**Response after childbirth**	**Response after 5 years**						**Two-sample test**	**Concordance rate**
	**Yes**		**No**		**I don’t remember**			
	**n**	**(%)**	**n**	**(%)**	**n**	**(%)**		
16. Did you release vocal outbursts during childbirth without excessively restraining them?								
Yes	440	(92.2)	33	(6.9)	4	(0.8)	< 0.001	87.4%
No	28	(62.2)	16	(35.6)	1	(2.2)		
17. Did you express the full range of your feelings during childbirth?								
Yes	433	(90.6)	42	(8.8)	3	(0.6)	< 0.001	84.0%
No	38	(80.9)	8	(17.0)	1	(2.1)		
18. Did you express yourself as you are during childbirth?								
Yes	444	(90.0)	46	(9.3)	4	(0.7)	< 0.001	85.7%
No	25	(78.1)	7	(21.9)	0	(0.0)		

**Table 6:** Correlation for the total score and factor scores of CBE-scale at baseline and 5 years postpartum stratified by subsequent childbirth and return to work

	**Total score**	**Factor score**			
		**"Happiness"**	**"Body sense"**	**"Discovery"**	**"Unaffected self expression"**
No stratified					
	0.44***	0.42***	0.46***	0.41***	0.19***
Subsequent childbirth					
Yes	0.46***	0.40***	0.52***	0.40***	0.23**
No	0.44***	0.43***	0.41***	0.41***	0.17**
Return to work					
Yes	0.44***	0.46***	0.47***	0.37***	0.27***
No	0.43***	0.37***	0.43***	0.43***	0.13*

*P > 0.05, **P > 0.01, ***P > 0.001 P-values for Spearman’s rank-order correlation test.

## Discussion

The most significant finding of this study was that women tended to remember the childbirth experience clearly at 5 years postpartum. There were significant differences on 16 out of 18 items on the CBE-Scale when responses to the same items on both surveys were compared. It means that there is a statistically significant relationship between the results of the initial survey and the results of the 5 years postpartum survey, which implies concordance of women’s memory of the item immediately after childbirth and women’s memory of the item after 5 years of childbirth. If women did not remember their childbirth experience clearly, the results of the initial survey and the results of the 5 years postpartum survey would not be the same and that there would not be observed statistically significant differences on this many items accordingly. The finding which women tend to remember the childbirth experience was particularly prominent among those who answered "yes" in response to CBE-Scale items at the baseline survey compared with those who did not. Many researchers, including Simkin, have shown that the childbirth experience has a powerful effect on women, with the potential for a permanent or long-term positive or negative impact [1]. Our results might explain the powerful effect of the childbirth experience on women’s lives if these memories are clearly remembered for prolonged periods.

We observed that women’s memories of the childbirth experience tend to become more positive at 5 years after childbirth. The percentage of responses to the same item on the CBE-Scale on both surveys that shifted from "no" to "yes" was more than 50% for 13 out of the 18 items. This tendency is consistent with previous findings that women’s memory of childbirth experience generally improves [6] and is in contrast to Waldenstrom’s findings [7]. The strength of our study lies in the fact that we evaluated women’s memory of the childbirth experience from various perspectives by using the items of a scale that was developed to evaluate the childbirth experience quantitatively. As various perspectives of the childbirth experience were evaluated, this study demonstrated that it was likely for women to forget unique experiences after childbirth such as "During childbirth, did you feel that you had no boundaries?".

However, this study has several limitations. First, the study population does not represent all Japanese women who delivered during the study period. The proportion of women who delivered at maternity homes in Japan was only 1.0% [10]. In contrast, the proportion of women who delivered at maternity homes in this study was 39.4%. The participants of this study were intentionally selected, as it was essential to recruit women whose childbirth experiences were both positive and negative to investigate the consistency of women’s memory for both aspects of the childbirth experience. Japanese maternity homes are well known for providing supportive and continuous care to women [11]. A previous study on Japanese maternity homes showed that women who deliver at maternity homes might have the chance to experience a dynamic spiritual state of their body leading them to a self-transforming experience. The 95% of women who delivered at maternity homes expressed full satisfaction with their experience; more than 60% mentioned they felt more confidence in themselves through their experience, and almost 50% felt a strong sense of compassion and sense of unity with the universe [12]. Most women who prefer to deliver at maternity homes actively seek the maternity home to enquire about the care that the maternity home provides. Therefore, the population of this study may be biased toward women who are more conscious about childbirth and maternal care compared to the entire Japanese population who delivered during the same period.

Second, women’s responses to both surveys may correspond by chance because dichotomous variables were used for each item of the CBE-Scale. The probability of correspondence by chance when using dichotomous variables is 50%. However, we believe that the use of the CBE-Scale was an optimal method for evaluating the childbirth experience in this study, because the scale was developed and evaluated for reliability and validity with the same participants. Furthermore, the analysis of our results also supports the conclusion that the correspondence of the answers to each item of the CBE-Scale did not occur by chance, because 16 out of 18 items of the CBE-Scale showed statistical differences. Indeed, the concordance rate on both surveys was more than 50% for all items of the CBE-Scale. In particular, the percentage of answers that corresponded among women who answered "yes" on the baseline survey was very high. It seemed that women who answered "no" on the baseline survey were more likely to be affected by recall bias than were those who answered "yes". In contrast, the percentage of women who answered "no" on both surveys was lower; the percentage of correspondence for most items on the CBE-Scale was less than 50%. Psychological studies have determined that emotionally arousing events are more accurately and vividly remembered [13]. This may help us understand the accuracy of memories of the childbirth experience, as childbirth has been described as one of the most emotional events for women. Emotionally arousing experience of childbirth would lead to the concordance of the women’s responses of CBE-Scale at baseline and those at 5 years postpartum.

Third, the effect of the difference between situations in which the participants answered the CBE-Scale at baseline and 5 years postpartum must be considered. In this study, participants answered the CBE-Scale just twice. We strove to collect data at baseline and follow-up in similar conditions, and thus the effect of potential biases such as maturation effects and mode effects were presumed to be minimal.

Memories of negative childbirth experiences were fewer than those of positive experiences. Nevertheless, it is important to acknowledge that many women have suffered negative experiences during childbirth, and there are often negative consequences of retaining the negative memories for a prolonged period. Some studies have revealed that negative childbirth experiences may lead to serious consequences such as post-traumatic stress disorder [3], maternal depression [14], opting for caesarean section at subsequent childbirth [15], and subsequent low fertility [16]. Previous research has shown that caregiver support during labor is related to childbirth experience [17-19]. A systematic review also revealed that women who had continuous intrapartum support were less likely to report dissatisfaction with their childbirth experiences [20]. Health care providers must consider the possibility that the care provided for women during the intrapartum period potentially affects both the childbirth experience, and encouragement of women after childbirth.

## Conclusion

We conclude that women remember their childbirth experience clearly at 5 years postpartum. This finding allows us to determine how women’s lives are impacted by childbirth. We hope that all women will remember childbirth as a good experience, regardless of the scientific evidence, although further research must be conducted to understand women’s memories of the childbirth experience and its impact on their lives.

## Competing interests

The authors declare that they have no competing interests.

## Authors’ contributions

KT, MN, TS and CS participated in design of the study, analysis and the draft of the manuscript. KT and MN carried out the data collection. CS supervised the whole study. All authors read and approved the final manuscript.

## Pre-publication history

The pre-publication history for this paper can be accessed here:

http://www.biomedcentral.com/1471-2393/14/221/prepub
